# Biomechanical comparison of five fixation methods in minimally invasive hallux valgus osteotomy: a three-dimensional finite element analysis

**DOI:** 10.3389/fmed.2025.1701147

**Published:** 2025-11-04

**Authors:** Xiaomei Li, Lan Zhang, Jiandong Wang, Yongjun Wang, Guangming Dai, Wei Jiang, Haoyan Zheng, Bo Feng, Weiqing Tian

**Affiliations:** Department of Orthopedics, The Third Affiliated Hospital of Inner Mongolia Medical University, Baotou, China

**Keywords:** hallux valgus, minimally invasive surgery, internal fixation, three-dimensional finite element method, biomechanics

## Abstract

**Objective:**

Although minimally invasive osteotomy for hallux valgus employs a variety of internal fixation methods, systematic biomechanical evidence evaluating the stability and strength of different fixation configurations is lacking. This study aimed to quantitatively compare the biomechanical properties of five internal fixation techniques using three-dimensional finite element analysis.

**Methods:**

Based on CT data of the foot of an adult female patient with moderate hallux valgus (HVA 27.6°, IMA 12.4°), a finite element model of the post-osteotomy state was constructed. The following fixation schemes were simulated: Group A: two 3.5 mm beveled metal screws; Group B: one 3.5 mm beveled metal screw; Group C: two 2.0 mm Kirschner wires; Group D: one 3.5 mm beveled metal screw and one 2.0 mm Kirschner wire; Group E: three 2.0 mm Kirschner wires. Comparison parameters included the maximum equivalent (Von-Mises) stress between the osteotomy fragment and the internal fixation, the maximum displacement of the osteotomy fragments in the X, Y, and Z axes, and the overall displacement of the internal fixation.

**Results:**

Under the same load: 1. Maximum stress of the osteotomy fragment: Group A (5.6824 MPa) < Group B < Group D < Group C < Group E (33.33 MPa); 2. Maximum stress of internal fixation: Group A (16.159 MPa) < Group D < Group B < Group C < Group E (238.68 MPa, with significant stress concentration); 3. Maximum displacement of the osteotomy fragment (X/Y/Z): Group E (4.2035/2.8512/7.1309 mm) < Group D < Group A < Group C < Group B (4.3251/3.2353/7.4102 mm); 4. Overall displacement of internal fixation: Group B (7.5284 mm) < Group D < Group C < Group A < Group E (7.9256 mm).

**Conclusion:**

1. Two 3.5 mm beveled screws (Group A) are the optimal configuration, combining low stress distribution (lowest stress on the osteotomy fragment and internal fixation) with high stability (moderate displacement); 2. Combined fixation (Group D) is a secondary option, but bone quality assessment is required (Kirschner wire fixation carries the risk of loosening); 3. Three Kirschner wires (Group E) are only suitable for low-load cases due to the risk of high stress concentration (238.68 MPa).

## 1 Introduction

Hallux valgus (HV) is the most common three-dimensional deformity of the human forefoot, characterized by progressive forefoot dysfunction caused by deviation of the first metatarsophalangeal joint complex (1–3). This deformity not only causes local pain, swelling, and difficulty wearing shoes (with an incidence of 23–35% in the adult population), but also increases the risk of secondary knee osteoarthritis due to gait abnormalities (4–6). Studies indicate (7) that both open and minimally invasive techniques are effective methods for correcting deformities, with third-generation minimally invasive surgery demonstrating potential advantages in improving the hallux valgus angle (HVA). Existing surgical approaches encompass multiple techniques, including percutaneous methods such as SERI, Bosch, and Reverdin-Isham; traditional open osteotomies like Scarf and Lapidus; and emerging intramedullary nail techniques. Among these numerous options, minimally invasive chevron-Akin (MICA) has become the mainstream surgical procedure for mild to moderate HV (mild deformity: 15° < HVA ≤ 20° and/or 9° < IMA ≤ 14°;moderate deformity: 20° < HVA ≤ 40° and/or 14° < IMA ≤ 20°) due to its advantages of minimal trauma and rapid recovery (8–10). Its core advantages include: 1. Biomechanical adaptability: special instruments enable large-scale translation of the metatarsal head ( > 5 mm), effectively correcting the intermetatarsal angle (IMA) to the normal range ( < 9°) (11, 12); 2. Advances in fixation technology: the third-generation MICA technique uses double-screw fixation—the proximal screw penetrates both cortices to lock the metatarsal head, and the distal screw engages a single cortex, significantly reducing displacement of the osteotomy end (13, 14); 3. Optimized surgical efficiency: guide wire pre-placement reduces intraoperative fluoroscopy frequency by approximately 40%, shortening the operation time. However, the current controversial fixation schemes focus on the following: 1. Doubts about the necessity of double screws: cadaver studies have shown that the failure strength of a single 4.0 mm screw (250 N) is sufficient for daily walking (peak load 300 N), but there is a risk of displacement (0.269 mm), which may affect early weight bearing (13, 15); 2. Limitations of alternative solutions: Although Kirschner wire or screw–Kirschner wire combined fixation is low-cost, the stress concentration of the Kirschner wire is significant ( > 150 MPa), and there is a risk of breakage. Although bandage fixation promotes secondary healing by achieving micro-motion (0.022–0.269 mm) through the “tendon-bone theory,” it requires frequent bandage changes (16); 3. Applicability to special populations: In patients with osteoporosis, Kirschner wire fixation is prone to loosening, and three Kirschner wires are recommended only for low-load cases due to their high risk of displacement (17). A current research bottleneck is the lack of systematic biomechanical evaluation data for minimally invasive fixation configurations, particularly quantitative comparisons of the following key parameters: 1. Maximum equivalent stress at the osteotomy site in different groups; 2. Stress distribution thresholds for different fixation devices; and 3. Overall displacement of the osteotomy fragment and internal fixation.

Thus, this study aimed to compare the biomechanical properties of five internal fixation schemes (including double screws, single screws, and a combination of Kirschner wires) in MICA osteotomy using high-precision finite element models. The objective was to quantify: maximum equivalent stress (predicting osteotomy fracture risk); overall internal fixation stiffness (assessing implant fatigue risk); triaxial displacement of the osteotomy site (assessing stability); and overall internal fixation displacement (reflecting torsional performance). By clarifying the mechanical properties and clinical indications of each scheme, this study will provide a basis for individualized fixation decisions and minimize the risk of metatarsal fracture and fixation failure (18–22).

## 2 Materials and methods

### 2.1 Study subjects

The model was constructed based on foot CT data from a 24-year-old female patient (height 158 cm; weight 52.5 kg) with moderate HV in a non-weight-bearing state. CT scan parameters included a slice thickness of 0.75 mm, a slice spacing of 0.75 mm, a matrix size of 512 × 512 pixels (Philips 16-slice spiral CT), and 321 DICOM-format images. Based on weight-bearing anteroposterior and lateral radiographs of the patient’s foot, the hallux valgus angle (HVA) was 27.6° and the first and second metatarsal angles (IMA) were 12.4°, meeting the clinical criteria for moderate deformity (20° < HVA ≤ 40° and/or 14° < IMA ≤ 20°) (8, 23). Patients were excluded from the study with a history of foot and ankle surgery, trauma, or arthritis. The study was approved by the Ethics Committee of Inner Mongolia Baogang Hospital (2024-MER-168).

### 2.2 Research methods

#### 2.2.1 Foot 3D model construction process

**FIGURE 1:**
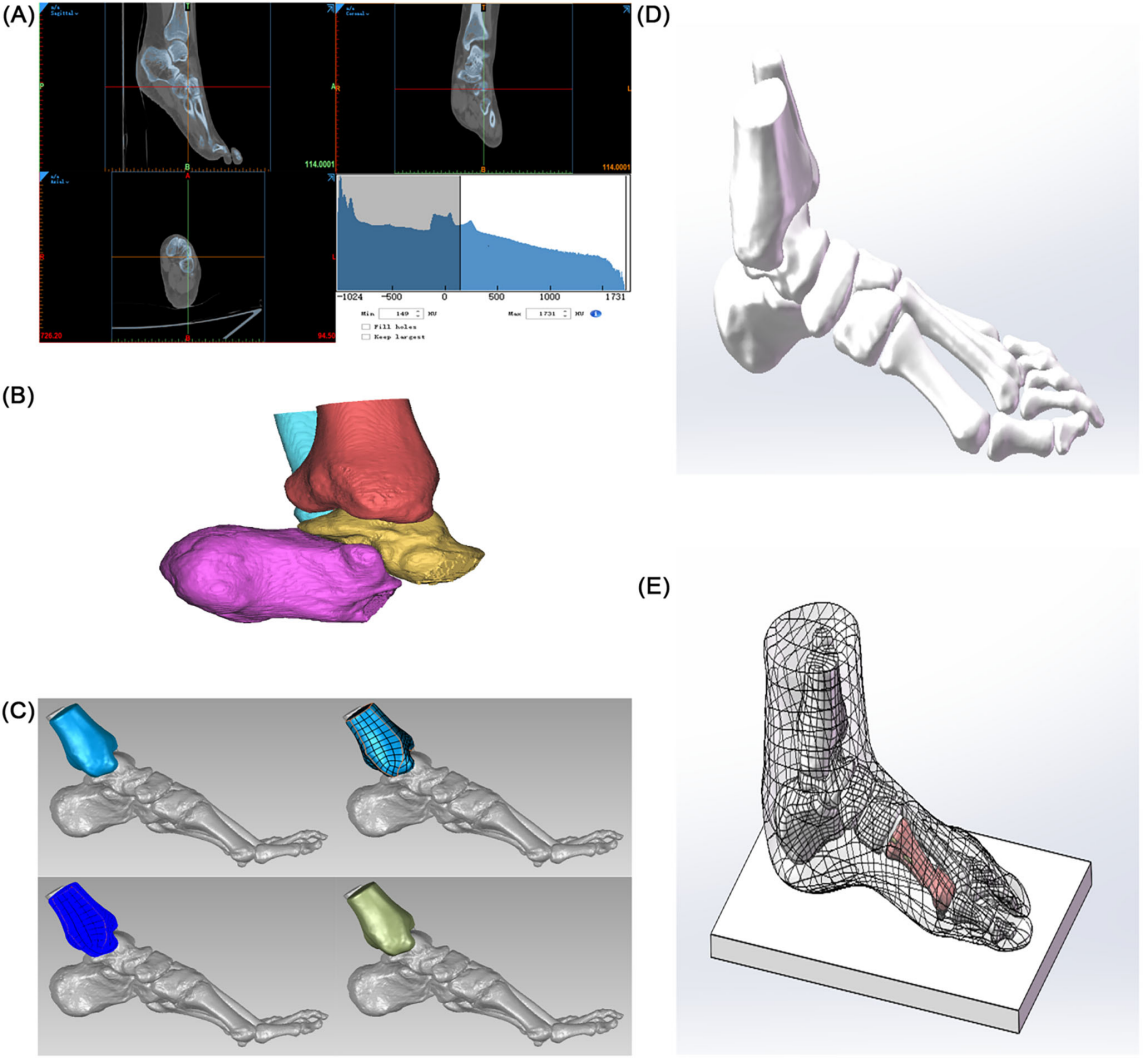
Construction of a finite element model of hallux valgus. **(A)** Preliminary 3D model of the foot. **(B)** Solidification of the 3D model. **(C)** Accurate surface (tibia as an example). **(D)** Skeletal assembly. **(E)** Simulated foot stationary on the ground.

Image Segmentation and Preliminary Modeling: DICOM-format CT data were imported into Mimics 21.0 (Materialise). A preliminary 3D foot model (STL format) was generated using threshold segmentation, region-growing, and bone/skin extraction. Surface Optimization and Anatomical Assembly: The STL file was imported into Geomagic 2021 for surface fitting and noise removal (accuracy < 0.1 mm). The optimized model was imported into SolidWorks 2022, where 30 bones (including the tibia and fibula, 7 tarsal bones, 5 metatarsal bones, 14 phalanges, and 2 sesamoid bones) and articular cartilage simulation (0.5 mm thickness) were completed. A skin soft tissue layer (2 mm thickness) and a rigid ground support surface were added to simulate a non-weight-bearing standing position (see Figure 1).

#### 2.2.2 Material properties

To better simulate the foot and avoid stress concentration, all solid parts were discretized into tetrahedral elements. The mesh sizes of bones and skin soft tissues, ground support and osteotomy blocks, and internal fixation were set to 3, 4, and 0.70 mm, respectively. In addition, local refinement was performed to adapt to the geometry of the contact area. The properties of biomaterials are critical parameters in finite element analysis. According to relevant literature (24), the material properties used in this study are represented by two parameters: elastic modulus (E) and Poisson’s ratio (ν) (see Table 1).

**TABLE 1:** Mechanical properties of physiological structures and internal fixation implants in the finite element model.

Parameters	Model materials					
	Cortical bone	Articular cartilage	Skin and soft tissue	Oblique metal screw	Kirschner wire	Ground support
Elastic modulus (E)	7,300	10	1.15	200,000	187,500	17,000
Poisson’s ratio (ν)	0.30	0.40	0.49	0.33	0.33	0.40

#### 2.2.3 Internal fixation design

**FIGURE 2:**
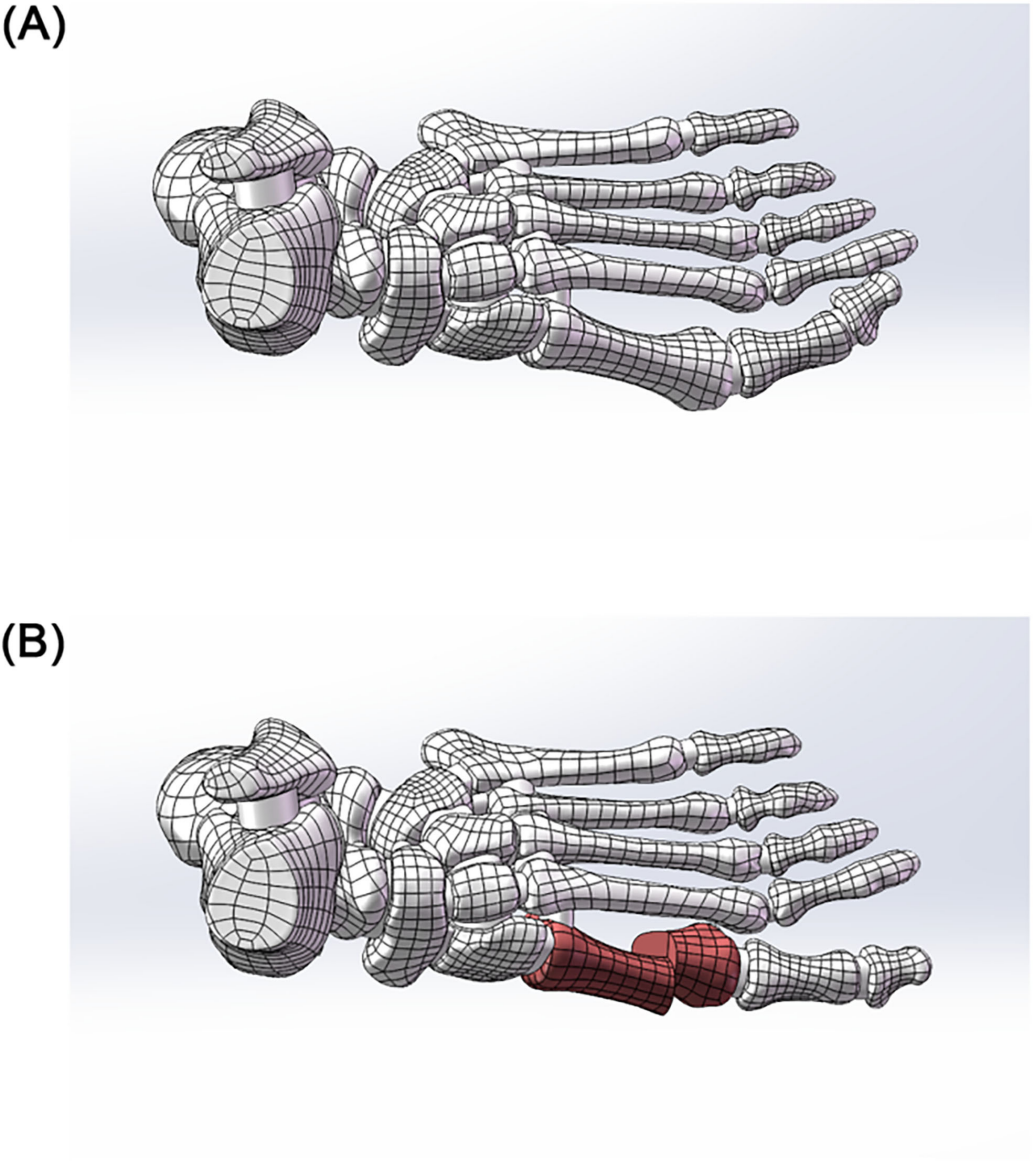
**(A)** 3D model before hallux valgus surgery. **(B)** 3D model after hallux valgus surgery.

**FIGURE 3:**
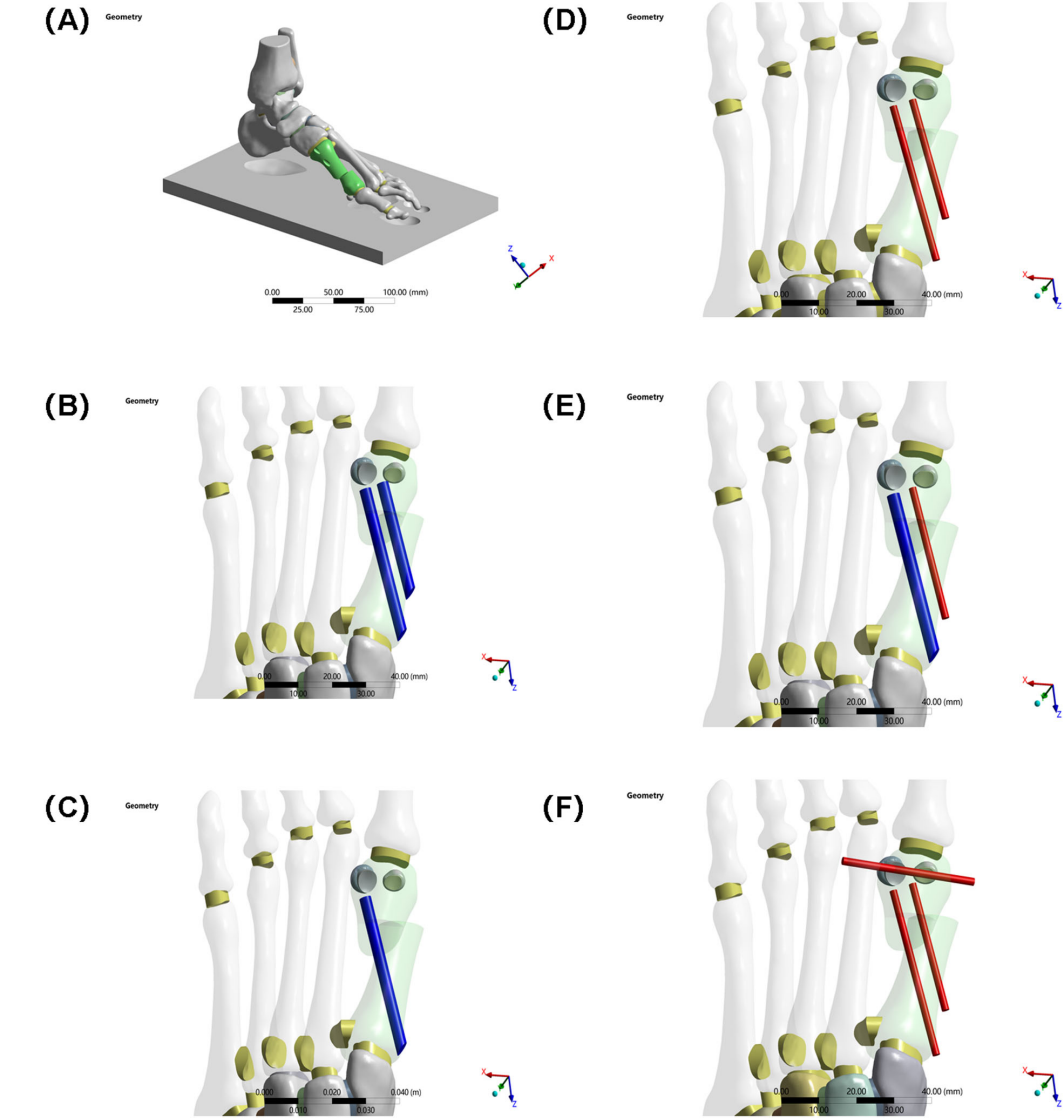
**(A)** Schematic diagram of the model. **(B)** Two 3.5 mm beveled metal screws. **(C)** One 3.5 mm beveled metal screw. **(D)** Two 2.0 mm Kirschner wires. **(E)** One 3.5 mm beveled metal screw and one 2.0 mm Kirschner wire. **(F)** Three 2.0 mm Kirschner wires.

Five fixation models were constructed (see Figures 2, 3):

Group A: Two 3.5 mm beveled metal screws (bicortical locking);

Group B: Single 3.5 mm beveled metal screw (single-point fixation);

Group C: Two 2.0 mm Kirschner wires (elastic fixation);

Group D: One 3.5 mm beveled screw and one 2.0-mm Kirschner wire (combined fixation);

Group E: Three 2.0 mm Kirschner wires (low-stiffness fixation).

Implantation specifications:

Standardized entry point: Medial to the base of the first metatarsal, penetrating the cortex according to the “in-out-in” principle;

Directional control: Parallel to the long axis of the metatarsal in the sagittal plane, and < 10° angle with the second metatarsal in the coronal plane.

#### 2.2.4 Boundary conditions and load settings

**FIGURE 4:**
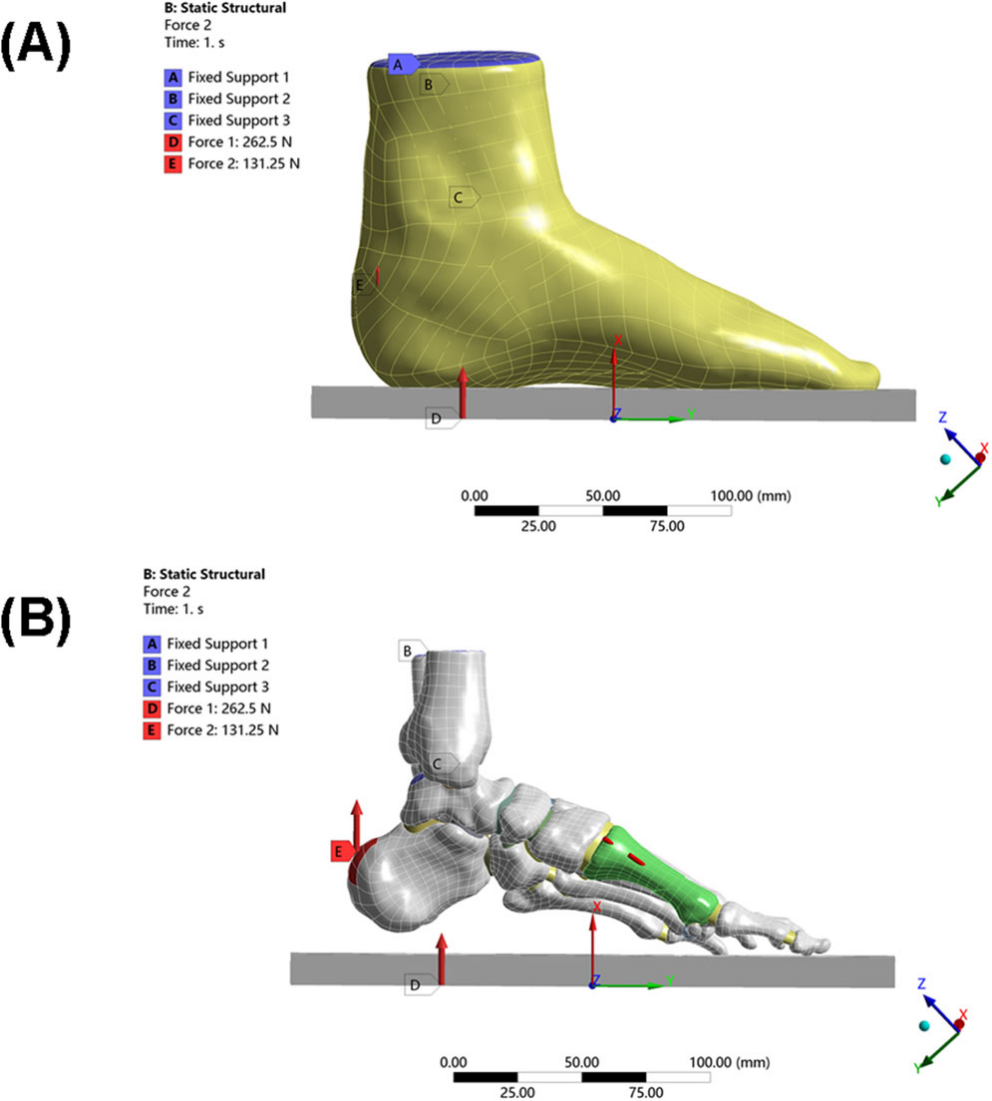
Loading conditions of the model. **(A)** Schematic diagram of overall foot loading. **(B)** Schematic diagram of local foot loading (including internal fixation).

Static neutral position simulation (see Figure 4):

Constraints: The skin bottom surface and the upper surface of the tibia/fibula were fully constrained;

The upper surface of the talus was constrained in rotational freedom (simulating the neutral ankle position);

Load application (25):

Ground reaction force: 262.5 N (50% of the patient’s body weight) applied vertically upward;

Achilles tendon load: 131.25 N (50% of the total load, simulating Achilles tendon tension during stance) applied vertically upward to the posterior end of the calcaneus.

#### 2.2.5 Biomechanical evaluation indicators

Fracture risk was assessed by the maximum equivalent stress of the osteotomy fragment, and fracture risk of the internal fixation was assessed by the maximum equivalent stress of the internal fixation device. The stability of the internal fixation system was assessed by maximum displacement of the osteotomy fragment in the X, Y, and Z axes and overall displacement of the internal fixation device.

## 3 Results

**FIGURE 5:**
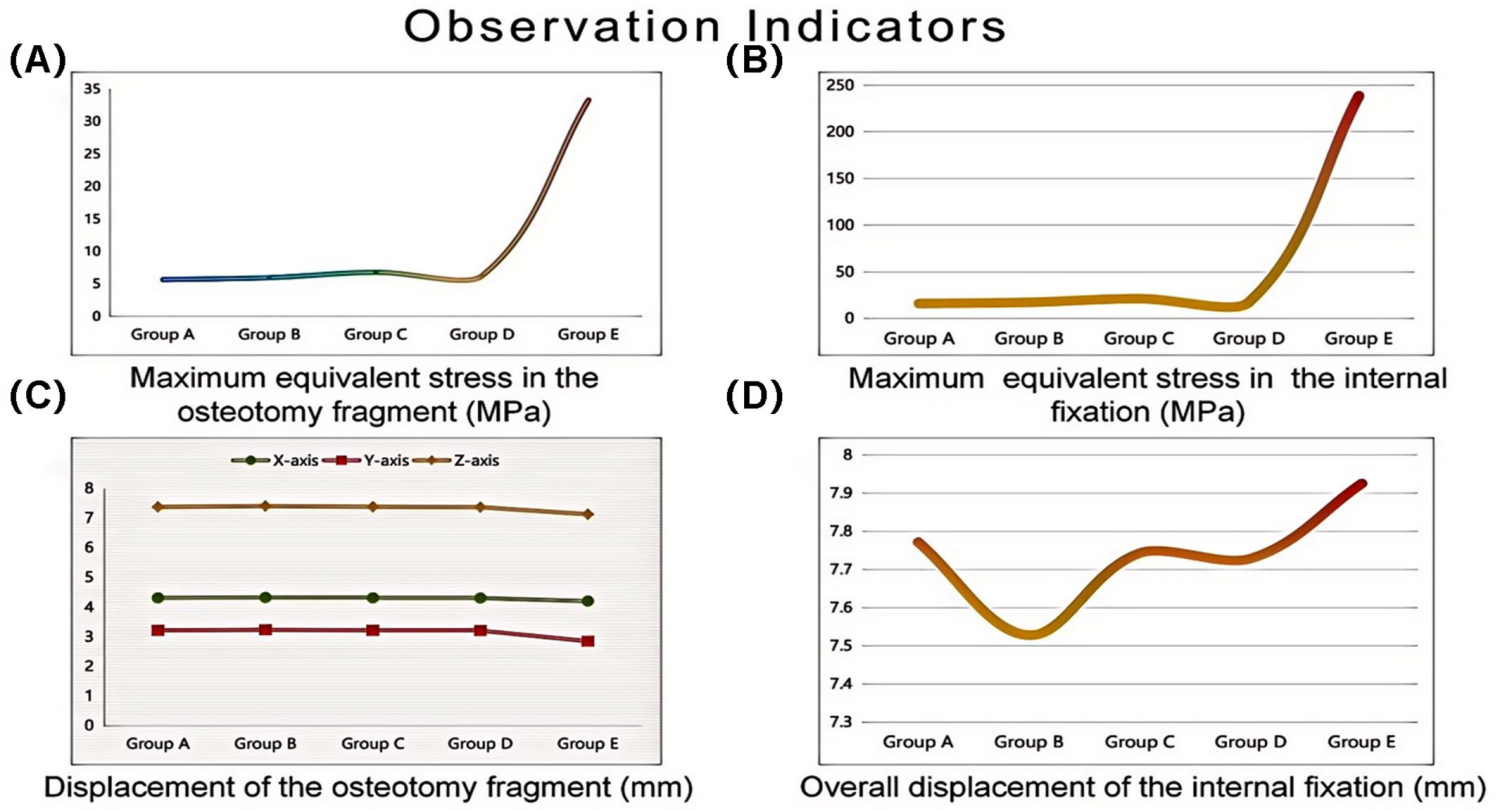
Finite element analysis of different internal fixation treatments in minimally invasive osteotomy. Invasive osteotomy. **(A)** Maximum equivalent stress in the osteotomy fragment (MPa). **(B)** Maximum equivalent stress in the internal fixation (MPa). **(C)** Maximum displacement of the osteotomy fragment (mm). **(D)** Overall displacement of the internal fixation (mm).

Under the aforementioned loading conditions, the maximum equivalent stresses of the osteotomy fragment and internal fixation, the maximum displacements of the osteotomy fragment in the X, Y, and Z directions, and the overall displacement of the internal fixation were calculated (see Table 2 and Figure 5).

**TABLE 2:** Finite element analysis of different internal fixation treatments in minimally invasive osteotomy.

Groups	Observation indicators					
	Maximum equivalent stress in the osteotomy fragment (MPa)	Maximum equivalent stress in the internal fixation (MPa)	Maximum displacement of osteotomy fragment (mm)			Overall displacement of the internal fixation (mm)
			X-axis	Y-axis	Z-axis	
Group A	5.6824	16.159	4.3118	3.2189	7.3794	7.7718
Group B	5.9929	17.512	4.3251	3.2353	7.4102	7.5284
Group C	6.8197	21.415	4.3125	3.2209	7.3847	7.7435
Group D	6.2728	17.496	4.3064	3.2102	7.3706	7.7292
Group E	33.33	238.68	4.2035	2.8512	7.1309	7.9256

### 3.1 Maximum equivalent stress of the osteotomy fragment

**FIGURE 6:**
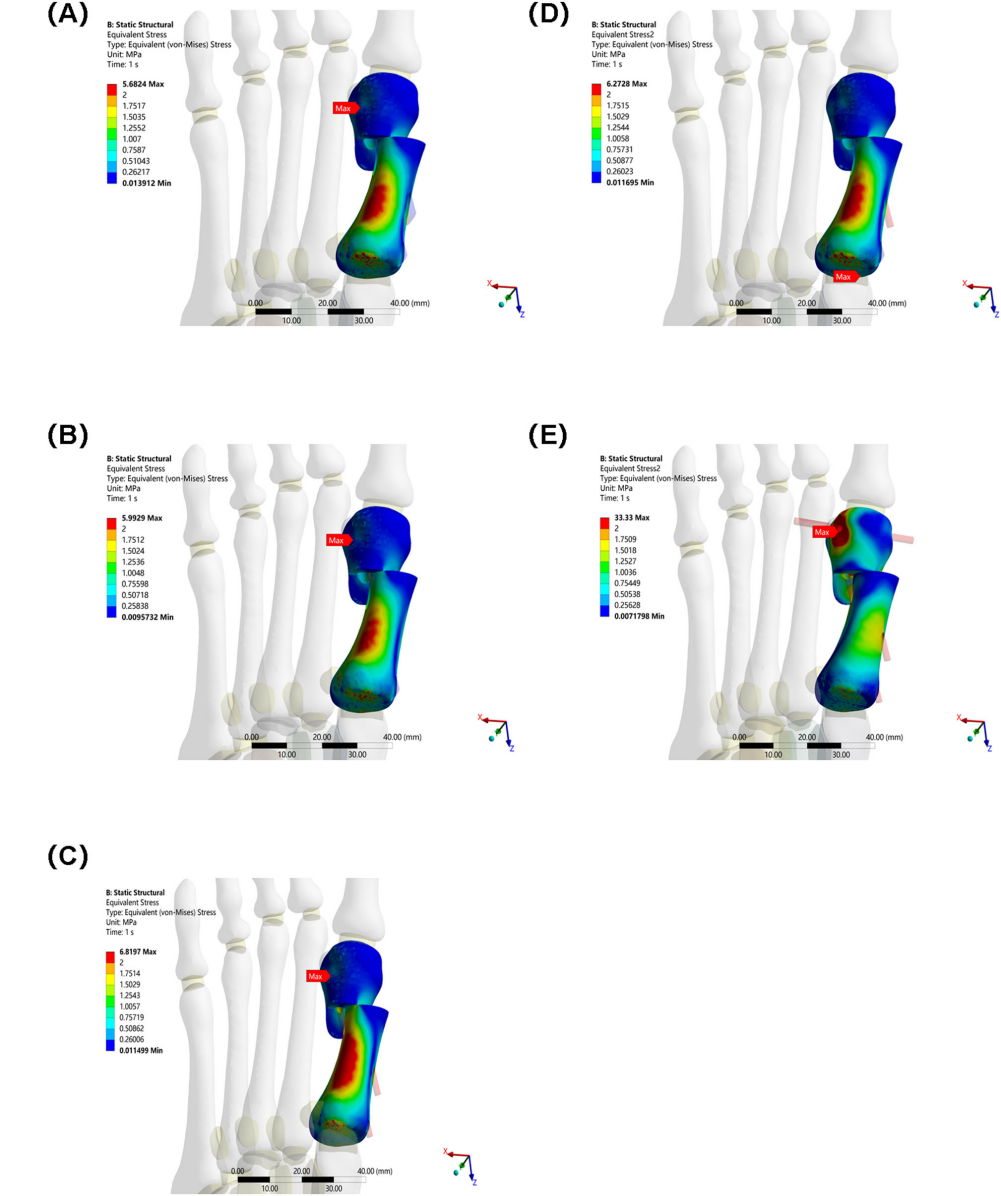
Maximum equivalent stress of osteotomy fragments in five groups under the same load. **(A)** Two 3.5 mm beveled metal screws. **(B)** One 3.5 mm beveled metal screw. **(C)** Two 2.0 mm Kirschner wires. **(D)** One 3.5 mm beveled metal screw and one 2.0 mm Kirschner wire. **(E)** Three 2.0 mm Kirschner wires.

The maximum equivalent stress of the osteotomy fragment in Group E (33.33 MPa) was significantly higher than that in Groups A–D (Group A: 5.6824 MPa, Group B: 5.9929 MPa, Group C: 6.8197 MPa, and Group D: 6.2728 MPa). This indicates that the osteotomy fragment in Group E was subjected to significantly higher mechanical loads under internal fixation, potentially increasing the risk of fragment fracture (see Figure 6).

### 3.2 Maximum equivalent stress of internal fixation

**FIGURE 7:**
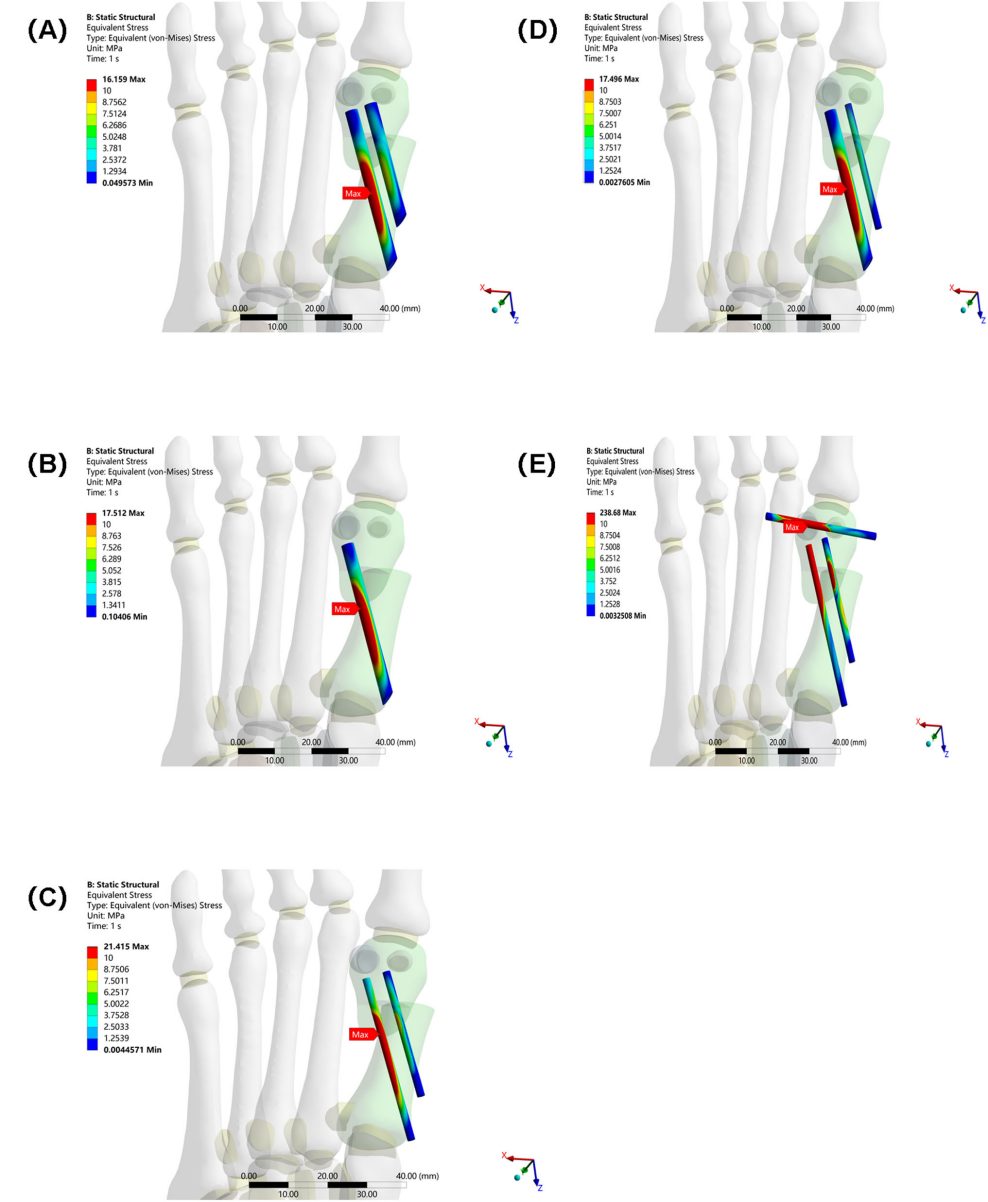
Maximum equivalent stress of internal fixation in five groups under the same load. **(A)** Two 3.5 mm beveled metal screws. **(B)** One 3.5 mm beveled metal screw. **(C)** Two 2.0 mm Kirschner wires. **(D)** One 3.5 mm beveled metal screw and one 2.0 mm Kirschner wire. **(E)** Three 2.0 mm Kirschner wires.

The maximum equivalent stress of the internal fixation in Group E (238.68 MPa) also far exceeded that of the other groups (Group A: 16.159 MPa, Group B: 17.512 MPa, Group C: 21.415 MPa, and Group D: 17.496 MPa). This suggests that the internal fixation in Group E was subjected to higher stress, making it more susceptible to failures such as fatigue and fracture. Among Groups A–D, the maximum equivalent stress at the interface of the osteotomy fragment and internal fixation in Group C was slightly higher, suggesting that mechanical transmission or the internal fixation material and structure differed from those in the other groups, resulting in a change in stress distribution (see Figure 7).

### 3.3 Osteotomy fragment maximum displacement in the X, Y, and Z directions

X-axis displacement: The values for each group were similar (Group A: 4.3118 mm, Group B: 4.3251 mm, Group C: 4.3125 mm, Group D: 4.3064 mm, Group E: 4.2035 mm). This indicates that in the X direction, the different internal fixations had little difference in their restraining effect on the osteotomy fragment, and the overall displacement patterns were similar.

Y-axis displacement: Group E (2.8512 mm) was lower than Groups A–D (Group A: 3.2189 mm, Group B: 3.2353 mm, Group C: 3.2209 mm, Group D: 3.2102 mm), possibly because the high stress concentration in Group E’s internal fixation constrained the Y-axis movement of the osteotomy fragment. The Y-axis displacement between Groups A–D fluctuated slightly, indicating similar mechanisms for internal fixation to regulate displacement in this direction.

**FIGURE 8:**
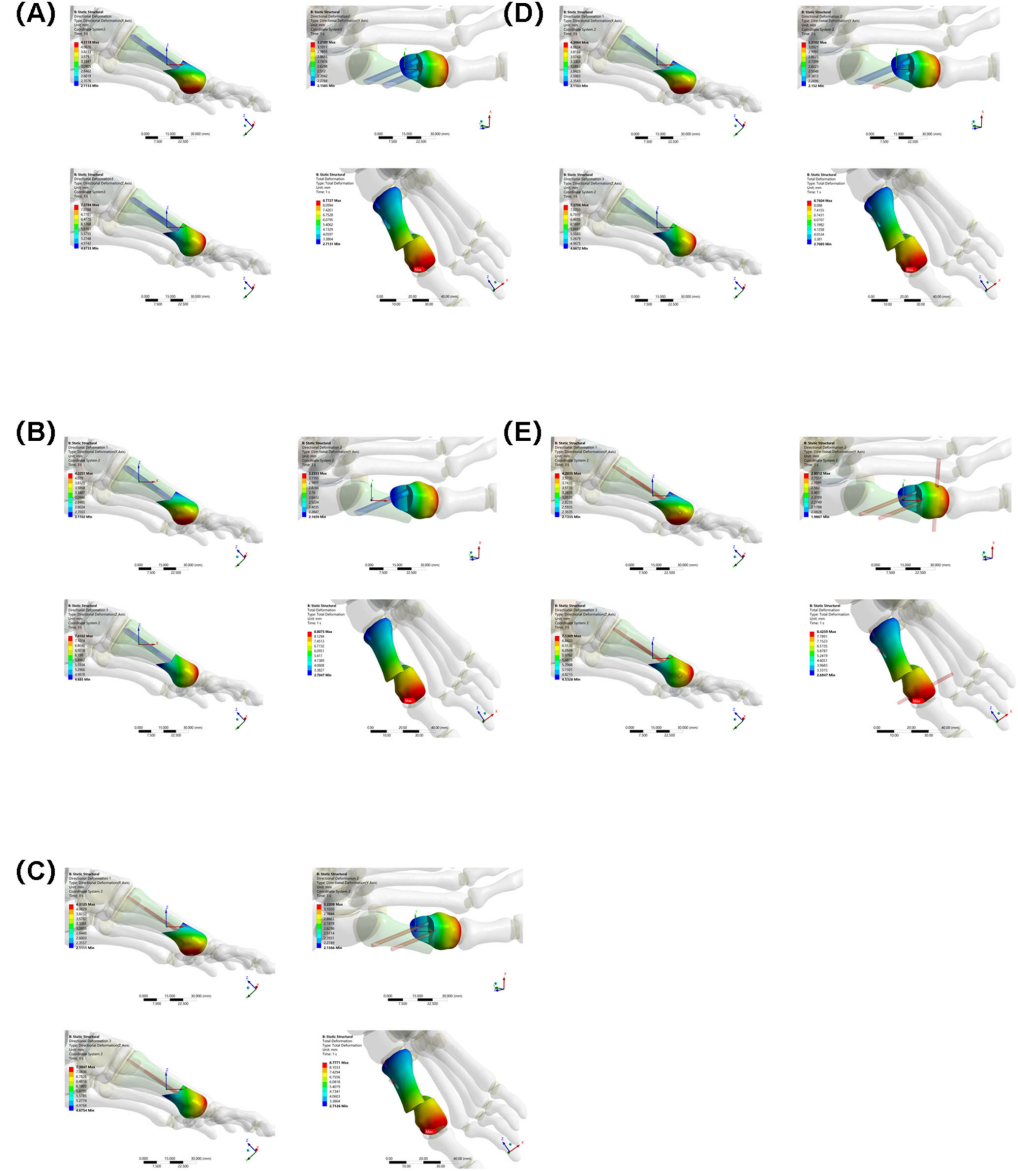
Maximum displacement of osteotomy fragments in five groups under the same load. **(A)** Two 3.5 mm beveled metal screws. **(B)** One 3.5 mm beveled metal screw. **(C)** Two 2.0 mm Kirschner wires. **(D)** One 3.5 mm beveled metal screw and one 2.0 mm Kirschner wire. **(E)** Three 2.0 mm Kirschner wires.

Z-axis displacement: The values differed slightly between the groups (Group A: 7.3794 mm, Group B: 7.4102 mm, Group C: 7.3847 mm, Group D: 7.3706 mm, Group E: 7.1309 mm), indicating that under different internal fixations, the Z-axis displacement of the osteotomy fragment was limited by the influence of internal fixation, and the overall displacement trend was relatively consistent (see Figure 8).

### 3.4 Overall displacement of internal fixation

**FIGURE 9:**
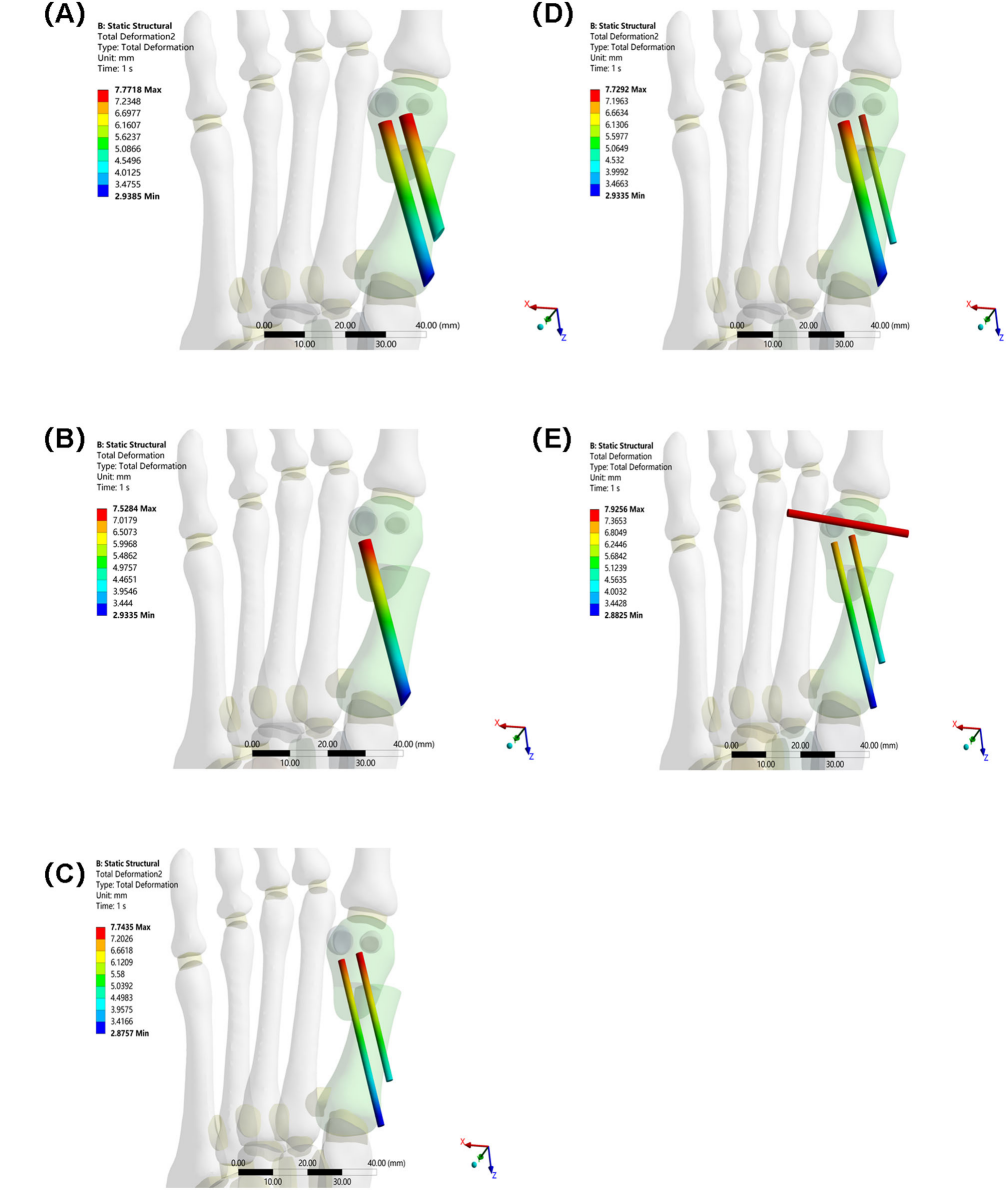
Overall displacement of internal fixation in five groups under the same load. **(A)** Two 3.5 mm beveled metal screws. **(B)** One 3.5 mm beveled metal screw. **(C)** Two 2.0 mm Kirschner wires. **(D)** One 3.5 mm beveled metal screw and one 2.0 mm Kirschner wire. **(E)** Three 2.0 mm Kirschner wires.

The overall displacement of the internal fixation in Groups A–D ranged from 7.5284 mm to 7.7718 mm (Group A: 7.7718 mm, Group B: 7.5284 mm, Group C: 7.7435 mm, and Group D: 7.7292 mm). These values are similar, indicating that the overall stability of the internal fixation in these four groups was similar, and they performed similarly in maintaining their position and transmitting mechanical loads. The overall displacement of the internal fixation in Group E was 7.9256 mm. Although not significantly different from Groups A–D, combined with its high-stress performance, Group E’s internal fixation still has some ability to control displacement under high loads. However, due to stress concentration, long-term stability may be affected (see Figure 9).

## 4 Discussion

HV is a common foot deformity in clinical practice. MICA has attracted significant attention due to its minimal trauma and rapid recovery (26, 27). In minimally invasive osteotomy, the optimal fixation scheme is influenced by the choice of internal fixation by orthopedic surgeons (16, 28, 29). Therefore, this study compared the stability, stress distribution, and displacement characteristics of five different internal fixation methods using three-dimensional finite element analysis on an adult female patient with moderate hallux valgus, aiming to provide a more scientific and rational basis for selecting internal fixation options.

In this study, Group A (bicortical screw fixation) demonstrated the best biomechanical properties. As shown in Table 2 and Figure 5, the maximum equivalent stresses of the osteotomy fragment and internal fixation were 5.6824 and 16.159 MPa, respectively (5.9929 MPa/17.512 MPa in Group B, 6.8197 MPa/21.415 MPa in Group C, 6.2728 MPa/17.496 MPa in Group D, and 33.33 MPa/238.68 MPa in Group E), all of which were the lowest among the five groups. Furthermore, the triaxial maximum displacements of the osteotomy fragment were 4.3118, 3.2189, and 7.3794 mm, respectively, and the overall displacement of the internal fixation was 7.7718 mm, all within reasonable ranges. These results suggest that bicortical screw fixation can evenly distribute vertical and horizontal shear forces across the first metatarsal cortex through multidirectional constraint, avoiding stress concentration at a single fixation point. This reduces micromotion at the osteotomy site and reduces the risk of osteotomy fragment refracture and internal fixation loosening and fracture. Selven and Lewis also confirmed this finding: in minimally invasive osteotomy, bicortical fixation provides superior biomechanical stability compared to intramedullary nail fixation, which is consistent with the results of this study. According to Figures 6, 7, the bicortical screw fixation in Group A demonstrated superiority in providing strong anti-rotational stability, which can effectively resist the shear force, torsional force and axial force encountered by the foot under weight-bearing conditions after surgery . In addition, bicortical screw fixation promotes close alignment of the osteotomy fragments and increases the contact area between the bone fragments, which not only promotes the formation of callus but also accelerates the process of bone healing. Therefore, bicortical screw fixation not only improves surgical stability, but also helps to shorten the patient’s recovery time and improve the patient’s quality of life. The conclusion of Ferreira et al. after a 2-year follow-up further supports this finding. They found that screw fixation in minimally invasive Chevron-Akin surgery can effectively correct HV and improve clinical and radiographic parameters . In summary, the bicortical screw fixation scheme in Group A, with its excellent stability, adaptability, and long-term reliability, meets fixation requirements, effectively promotes foot function recovery, and significantly reduces the risk of recurrence. In addition to Group A, other groups also demonstrated good biomechanical properties, such as Group D.

Group D (one 3.5 mm cortical screw combined with one 2.0 mm Kirschner wire) is an effective alternative to Group A, showing the second-highest equivalent stress of osteotomy blocks and internal fixation after Group A. The results of this study showed that the biomechanical properties of Group B (single 3.5 mm cortical screw fixation) were slightly inferior to those of Group A, while the mechanical properties of Group D complemented those of Group A. The core of this result lies in the synergistic mechanical effect of “screw + Kirschner wire”: the 3.5 mm screw mainly provides the ability to resist vertical loads, while the 2.0 mm Kirschner wire supplements the ability to resist horizontal shear and torsional loads. This combination not only retains the rigid support advantage of screw fixation, but also reduces damage to the surrounding trabeculae by reducing the number of screw implants, achieving a balance between stability and minimal invasiveness. In addition, Jung, H.G.’s study showed that in proximal metatarsal osteotomy, the combined use of Kirschner wire and screw fixation (KWS group) can significantly improve fixation stability and reduce the risk of postoperative deformity recurrence compared with the use of Kirschner wire fixation alone (KW group) . However, for patients with osteoporosis, there is a risk of Kirschner wires being dislodged, so it is important to pay attention to the patient’s bone condition during clinical use, which was also confirmed by Hayashi’s study . While the bone displacement difference between Groups A and D (less than 0.2 mm) appears statistically insignificant, such minor variations may carry clinical significance regarding long-term stability and bone union quality. Even subtle movements of 0.1–0.2 mm under sustained physiological loads can indirectly influence healing duration and patients’ confidence in early weight-bearing through effects on initial callus formation and remodeling processes, although their direct impact may be less pronounced compared to the biocompatibility of internal fixation methods and surgical techniques themselves . It is worth noting that although the biomechanical properties of Groups A and D were better, the finite element analysis results of Group E (fixed with three Kirschner wires) suggested a worst-case fixation method that requires special attention.

The results of Group E showed a paradoxical feature—high stability and high fracture risk coexist. Although the maximum displacement of its osteotomy fragment was the smallest among all groups, showing strong immediate stability, the maximum equivalent stress of its osteotomy fragment and internal fixation was significantly higher than that of other groups, 5.8 times and 14.7 times that of Group A, respectively, indicating severe stress concentration. The underlying biomechanical mechanism for this “low-displacement, high-stress” paradox lies in the rigid frame formed by the three Kirschner wires, which effectively restricts macroscopic displacement of the bone fragment under initial loading but simultaneously alters the normal load transmission pathway. The transverse Kirschner wire crossing the first and second metatarsals creates a significant “lever fulcrum,” preventing effective dispersion of the load along the longitudinal axis of the first metatarsal. This leads to extreme stress concentration (238.68 MPa) at the interfaces between the Kirschner wires and bone holes, as well as at the osteotomy site . Highly consistent with clinically observed failure modes such as Kirschner wire breakage, bone resorption, and fixation loosening. Under high-cycle cyclic loading, this stress concentration area is highly prone to initiating metal fatigue microcracks and causing local resorption of surrounding bone tissue, ultimately manifesting as radiographic loosening or clinical fixation failure.

Clinically, the high-stress pattern in Group E aligns with documented complications of multi-K-wire fixation. Pin tract infection remains prevalent, with approximately 7% of hand and wrist fracture patients requiring oral antibiotics, early pin removal, or reoperation due to infection . Although generally manageable, these infections pose a tangible clinical burden. The transverse placement of K-wires between the first and second metatarsals risks injuring intermetatarsal ligaments and neurovascular structures, while potentially restricting physiological micromotion of the first ray. Additionally, as with any fixation in this region, both K-wires and screws carry a risk of joint penetration, which may lead to persistent pain, cartilage damage, and functional impairment . When combined with the significant stress concentration observed in our finite element analysis, these clinical findings strongly suggest that multi-K-wire configurations should be reserved for carefully selected low-demand cases where potential benefits clearly outweigh the documented risks.

This study has several limitations that should be considered when interpreting the results. First, the findings are derived from a single finite element model based on one patient’s anatomy. While this allows for a controlled comparison of fixation methods, it limits the generalizability of the absolute values to a broader population with varying bone quality and anatomical morphologies. The model aimed to replicate the mechanical environment following osteotomy and fixation, and the comparative trends observed are informative, but the results require validation against multi-patient models or experimental data. Second, the model was constructed from non-weight-bearing CT scan data. This choice was made to obtain precise bony geometry without the confounding effects of soft tissue deformation and positional changes under load, which is a common approach in foundational finite element studies of the foot. However, this means the model does not capture the functional joint alignments and contact conditions under physiological loading, potentially influencing the simulated stress distribution and displacement outcomes. Future models incorporating weight-bearing CT or simulated load-deformation relationships could enhance physiological relevance. Third, the simulation employed a static load condition representing a portion of the body weight during single-leg stance. While this provides a standardized basis for comparing fixation constructs, it does not account for the dynamic, multi-directional loads (e.g., shear, torsion, cyclic fatigue) experienced during gait. Consequently, the model might underestimate the peak stresses and potential failure risks associated with long-term cyclic loading. Finally, this biomechanical study provides evidence on the stability and strength of different fixation configurations but does not constitute clinical evidence of superiority. The generalizability of these finite element results to other surgical techniques that include Akin osteotomy may be limited . Future research should build on this foundation by incorporating dynamic gait simulations, conducting cadaveric experiments for validation, and pursuing long-term clinical follow-up studies to correlate biomechanical properties with patient outcomes. This will provide more comprehensive and reliable theoretical support and practical guidance for minimally invasive surgery for HV, provide more personalized treatment for patients, and promote postoperative functional recovery and improved quality of life.

## 5 Conclusion

The choice of internal fixation for minimally invasive hallux valgus osteotomy requires comprehensive consideration of biomechanical stability, surgical trauma, individual patient conditions, and medical costs. This finite element analysis, based on a single patient-specific model, provides quantitative biomechanical insights but cannot be directly generalized to all patient populations. Within the context of this computational model, double screw fixation (Group A) demonstrated favorable biomechanical performance, while combined fixation (Group D) emerged as a potential alternative. Kirschner wire fixation, particularly the three-wire configuration (Group E), exhibited high stress concentration and should be applied with strict adherence to specific clinical indications. The findings indicate relative trends in stability and stress among the fixation methods rather than establishing definitive clinical superiority.

## Data Availability

The original contributions presented in the study are included in the article/supplementary material, further inquiries can be directed to the corresponding authors.
